# Direct Experimental Evidence for Substrate Adatom Incorporation into a Molecular Overlayer

**DOI:** 10.1021/acs.jpcc.2c01432

**Published:** 2022-04-19

**Authors:** Philip J. Mousley, Luke A. Rochford, Paul T. P. Ryan, Philip Blowey, James Lawrence, David A. Duncan, Hadeel Hussain, Billal Sohail, Tien-Lin Lee, Gavin R. Bell, Giovanni Costantini, Reinhard J. Maurer, Christopher Nicklin, D. Phil Woodruff

**Affiliations:** † Diamond Light Source, Harwell Science and Innovation Campus, Didcot OX11 0DE, U.K.; ‡ Chemistry Department, University of Birmingham, University Road, Birmingham B15 2TT, U.K.; § Department of Materials, Imperial College, London SW7 2AZ, U.K.; ∥ Department of Physics, University of Warwick, Coventry CV4 7AL, U.K.; ⊥ Department of Chemistry, University of Warwick, Coventry CV4 7AL, U.K.

## Abstract

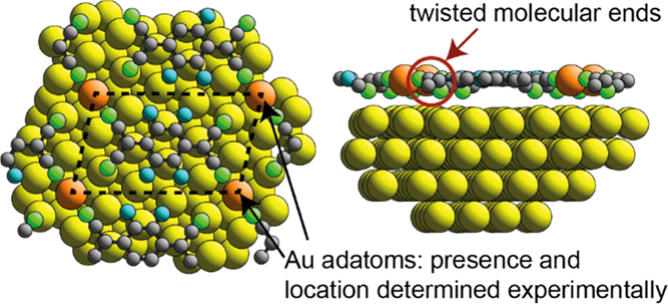

While the phenomenon of metal substrate adatom incorporation into molecular overlayers is generally believed to occur in several systems, the experimental evidence for this relies on the interpretation of scanning tunneling microscopy (STM) images, which can be ambiguous and provides no quantitative structural information. We show that surface X-ray diffraction (SXRD) uniquely provides unambiguous identification of these metal adatoms. We present the results of a detailed structural study of the Au(111)-F_4_TCNQ system, combining surface characterization by STM, low-energy electron diffraction, and soft X-ray photoelectron spectroscopy with quantitative experimental structural information from normal incidence X-ray standing wave (NIXSW) and SXRD, together with dispersion-corrected density functional theory (DFT) calculations. Excellent agreement is found between the NIXSW data and the DFT calculations regarding the height and conformation of the adsorbed molecule, which has a twisted geometry rather than the previously supposed inverted bowl shape. SXRD measurements provide unequivocal evidence for the presence and location of Au adatoms, while the DFT calculations show this reconstruction to be strongly energetically favored.

## Introduction

It is now recognized that molecular adsorption on metal surfaces often leads to significant modification of the structure of both the adsorbed molecule and the metal surface. One example of this metal surface modification is the reported adsorption-induced incorporation of metal adatoms from the bulk into the molecular overlayer, often forming two-dimensional metal organic frameworks (*e.g*.^1^). This effect is also believed to play a key role in surface-assisted Ullmann coupling reactions (*e.g*.^2^). Despite several reports of this phenomenon, there are no quantitative experimental determinations of these surface structures. The present evidence for the phenomenon comes mostly in the form of atomic-scale protrusions seen in constant tunneling current scanning tunneling microscopy (STM) images, interpreted as being due to these metal adatoms. However, STM provides no identification of the atomic species leading to such features, and indeed there is even no totally reliable correlation between atomic-scale protrusions seen in STM images and the positions of surface atoms.^3,4^ In a few studies, the assignment of these features to metal adatoms is supported by density functional theory (DFT) simulations of the STM images, based on the simple Tersoff–Hamman approach,^5^ which takes no account of the role of the tunneling tip in the imaging process.

In recent years, the experimental technique that has provided most of the quantitative information on the structure of metal–organic interfaces, and particularly on the height of the adsorbed molecule above the surface, is normal incidence X-ray standing waves (NIXSW).^6^ This technique generates quantitative information on the location of the different constituent atoms of an adsorbed molecule relative to the underlying substrate, by monitoring their core-level photoemission as the X-ray standing wave, established at a Bragg reflection, is swept through the crystal. This information is element-specific due to the characteristic photoelectron binding energies of these core levels, while chemical shifts in these energies make it also possible to determine the distinct local sites of atoms of the same element in different chemical bonding states within the molecule. However, NIXSW is not able to distinguish between emission from metal adatoms and from the vastly larger number of atoms of the same metal in the underlying substrate; any associated chemical shifts are too small to be exploited, even using detection at grazing emission angles. The technique is thus ‘blind’ to metal adatoms, although their presence may be inferred from their impact on the resulting molecular conformation, as in the case of 7,7,8,8-tetracyanoquinodimethane (TCNQ) adsorbed on Ag(111).^7^

One technique that can be expected to provide direct evidence of the presence and location of metal adatoms is surface X-ray diffraction (SXRD). Although the general technique of XRD is not explicitly element-specific, the scattering cross sections scale as the square of the atomic number, *Z*. One consequence of this is that SXRD is regarded as unsuitable to determine the structure of adsorbed molecules in which all of the constituent atoms have low *Z* (C, N, O, H). However, if the underlying metal has a much higher *Z* (*e.g.* Cu, Ag, Au), metal adatom incorporation will lead to the intensities of diffracted beams arising from the overlayer periodicity being dominated by scattering from the adatoms in this layer. This sensitivity to the location of high-*Z* elements is the basis of the ‘heavy atom’ method of solving complex macromolecular crystal structures with XRD and is also relevant to the related techniques of isomorphous replacement and multiwavelength anomalous dispersion XRD (MAD) (*e.g*.^8^).

Here, we present the results of an investigation to demonstrate and explore the application of SXRD to a two-dimensional (2D) metal–organic overlayer believed to be created by metal adatoms. The model system we have chosen to investigate is the fully fluorinated version of TCNQ, F_4_TCNQ, adsorbed on Au(111),^9^ for which published STM results, supported by DFT image simulations, have been interpreted as evidence for Au adatom incorporation into the molecular overlayer. There have also been both earlier^10^ and later^11^ reports of DFT calculations of F_4_TCNQ adsorption on Au(111), but these take no account of any possible surface reconstruction. The underlying motivation for investigating this, and closely related adsorption structures, is the need to understand the critical role that metal–organic interfaces play in determining the electronic properties of organic devices. TCNQ and F_4_TCNQ have attracted considerable interest as additives in organic electronics, due to their strong electron acceptor properties, resulting in several model studies of these species adsorbed on coinage metal surfaces (*e.g*.^7,9−17^), while F_4_TCNQ adsorption and incorporation into Ag surfaces have been shown to produce high work function electrodes for organometallic growth.^18^

Our comprehensive experimental and theoretical investigation of the ordered Au(111)-F_4_TCNQ adsorption phase is based on initial characterization with STM, low-energy electron diffraction (LEED), and X-ray photoelectron spectroscopy (XPS) using incident synchrotron radiation at a photon energy of 2.2 keV. Complementary quantitative structural information has then been obtained from NIXSW and SXRD. We have also undertaken a new investigation of the energetics and structure through dispersion-corrected DFT calculations. Although the earlier investigation of this system based on STM^9^ included some DFT calculations, these did not contain any corrections for van der Waals interactions that are known to strongly influence the adsorption height of these molecules on metal surfaces. Moreover, this earlier publication reported no quantitative structural results. Our results provide a completely consistent experimental and theoretical picture of the structure of F_4_TCNQ adsorption on Au(111), with SXRD measurements providing unequivocal experimental evidence for the presence and location of Au adatoms in the overlayer.

## Methods

### Experimental Details

Initial characterization of the conditions for the preparation of the ordered adsorption phase of F_4_TCNQ on Au(111) was performed in a combined LEED/STM ultrahigh vacuum (UHV) surface science chamber at the University of Warwick. The Au(111) sample was subjected to *in situ* cleaning by cycles of argon ion bombardment and annealing to achieve constant current STM images and LEED patterns showing the characteristic ‘herring-bone’ reconstruction of the clean surface, which is lifted by adsorption of F_4_TCNQ. All imaging and LEED patterns were obtained at room temperature, while the LEED patterns were recorded using a low incident beam current microchannel-plate-amplified MCP-LEED optics. All STM images were plane-corrected and flattened using the open-source image-processing software Gwyddion.^19^

Further characterization of the surface by SXPS, together with quantitative structural information from NIXSW (also recorded at room temperature), was obtained using the UHV surface science endstation at beamline I09 of the Diamond Light Source.^20^ Sample cleaning and F_4_TCNQ deposition using an organic molecular beam epitaxy source were performed using the same methods of the initial characterization as at the University of Warwick, with a similar MCP-LEED optics providing a direct cross-reference of the successful formation of the  ordered F_4_TCNQ adsorption phase. All measurements were made using the crystal monochromator branch of this beamline that delivers ‘hard’ X-rays with energies greater than ∼2 keV. The endstation chamber is equipped with a VG Scienta EW4000 concentric hemispherical electron energy analyzer with an extra-wide (±30°) angle acceptance mounted at 90° to the incident beam, which is used to collect core-level photoemission spectra for both of these techniques.

NIXSW measurements were taken by stepping the photon energy of the X-ray beam at normal incidence to the surface through the (111) Bragg reflection of the Au substrate at a nominal energy of 2636 eV, recording the photoemission spectra around the C 1s, N 1s, and F 1s emission at each step. These spectra were fitted by the chemically shifted components and the variation of intensity of each component as a function of photon energy was then fitted by the standard NIXSW formulae (taking account of the influence of backward–forward asymmetry in the photoemission angular dependence)^6^ to yield optimum values for the two key structural parameters, the coherent fraction, *f*, and the coherent position, *p*. Nondipolar effects in the angular dependence of the high-energy photoemission were corrected as previously described^6^ using values of the backward–forward asymmetry parameter, *Q*, derived from the published theoretical calculations.^21^ It was assumed that these nondipolar effects in the measurements using a wide angular range of emission detection could be modeled by a mean value of the polar emission angle, θ, defined as the angle between the photon polarization and the photoelectron detection direction. Due to the strongly attenuated signal coming from near 90° grazing emission angles, a θ of 18° was used.

SXRD measurements were made using the UHV surface science endstation of beamline I07 of the Diamond Light Source.^22^ Sample preparation followed the same methods used at the University of Warwick and at beamline I09 and the formation of the required F_4_TCNQ ordered overlayer phase was checked with a standard LEED optics. An incident photon energy of 11.4 keV was used, chosen to be below the L-edges of Au, thereby avoiding a significant fluorescence background from the substrate, with a grazing incidence angle of 0.3°.

### DFT Calculations

DFT calculations were performed with the FHI-aims package^23^ and a GGA-PBE functional^24^ was used to evaluate exchange correlation. Dispersion interactions were modeled using the Tkatchenko–Scheffler vdW^surf^ method (PBE + vdW^surf^).^25^ The adsorption structure was modeled as a periodically repeated cell comprising a single  unit mesh on Au(111) containing a single F_4_TCNQ molecule and either one Au adatom or no adatoms. The Au(111) surface was modeled as a slab consisting of four atomic layers and separated from its periodic image by a vacuum gap exceeding 60 Å. The coordinates of the atoms in the bottom two layers of the Au slab were constrained to the bulk truncated structure of Au and the positions of all other atoms in the simulation cell were relaxed. During optimization, we neglected long-range dispersion interactions between Au atoms and used the default “tight” basis set definition within FHI-aims. The Brillouin zone was sampled with an 8 × 8 × 1 Monkhorst-Pack^26^ k-grid and the geometries were optimized to below a force threshold of 0.025 eV/Å. A dipole correction was employed in all cases. All DFT calculation inputs and outputs are freely available and can be found as a dataset in the NOMAD repository via https://dx.doi.org/10.17172/NOMAD/2022.01.31-1.

## Results and Discussion

### Experimental Surface Characterization

**Figure 1:**
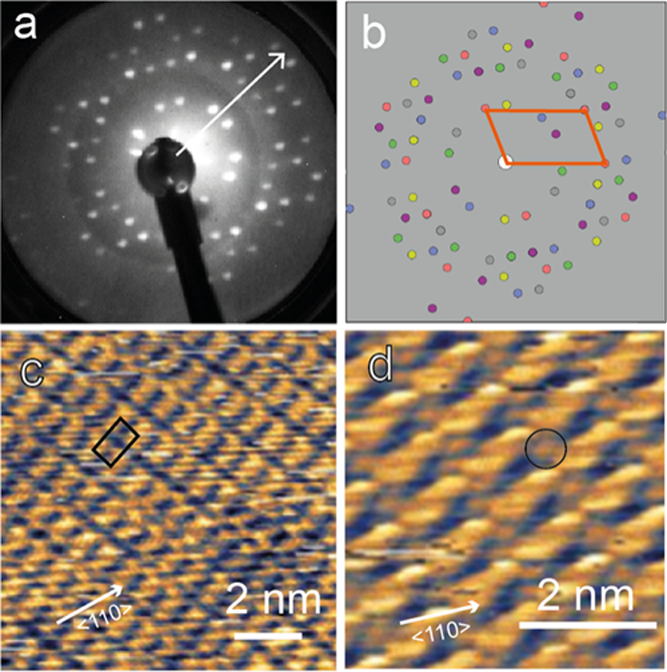
(a) LEED pattern recorded from the Au(111)-F_4_TCNQ surface at an electron energy of 24.5 eV. (b) Simulation of the LEED pattern based on the matrix, with diffracted beams from all rotational and mirror reflection domains shown in different colors. The reciprocal unit mesh of one of these (orange) is superimposed. (c) 10 nm × 10 nm constant current STM image of this surface with a unit mesh superimposed. (d) 5 nm × 5 nm STM image of a different area showing some evidence (circled) of features previously attributed to Au adatoms. The arrows correspond to a ⟨110⟩ direction on the surface. STM imaging conditions (sample bias and current): (c) +1.25 V, 250 pA and (d) −1.00 V, 75 pA.

Deposition of F_4_TCNQ onto the Au(111) surface held at room temperature, from an organic molecular beam deposition source, led to the formation of the  ordered phase previously identified by STM^1^ and LEED.^27^Figure 1a shows the resulting LEED pattern, obtained using a low incident current multichannel-plate amplified optics (MCP-LEED). Due to the low symmetry of the unit mesh of this phase, the pattern is a sum of the patterns from multiple domains related by the rotational and mirror symmetry elements of the substrate. Figure 1b shows a simulation, using the LEEDpat program^28^ of the LEED pattern to be expected for the  mesh, with diffracted beams from different domains shown in different colors. The agreement with the experimentally recorded pattern is excellent confirming the validity of the unit cell assignment. Figure 1c shows a typical constant tunneling current STM image of this surface. The spatial resolution of this STM image, recorded at room temperature, is undoubtedly inferior to the exceptional resolution of one of the images presented in the earlier paper by Faraggi et al.^9^ that was recorded at 5 K, but the same periodicity is clear. In very few images, such as Figure 1d, there is possible evidence of the protrusions (one is circled) attributed in this earlier study to the presence of Au adatoms.

**Figure 2:**
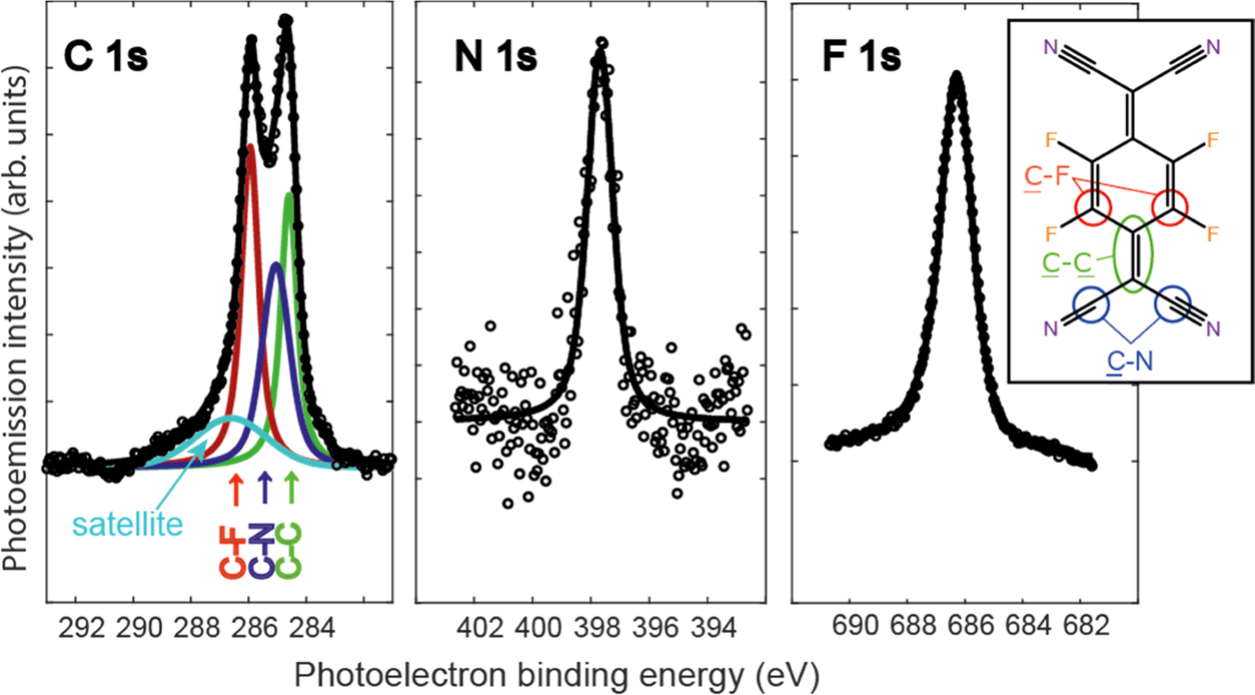
XPS C 1s, N 1s, and F 1s spectra from the Au(111)-F_4_TCNQ surface recorded at a photon energy of 2.2 keV (open circles), showing single-component fits (continuous lines) to the N 1s and F 1s spectra and fits to the chemically shifted components of the C 1s spectrum.

Figure 2 shows XP spectra in the energy ranges of the C 1s, N 1s, and F 1s emissions; the C 1s spectrum shows the chemically shifted components associated with CF, CC, and CN bonding, although the CN component is not clearly resolved in the raw spectrum. This spectrum is similar to one reported by Hählen et al. from a nominal monolayer of F_4_TCNQ on Au(111)^29^ and quite different from the spectrum resulting from multilayer deposition presented by these authors. The N 1s and F 1s spectra are fitted by single symmetric peaks, although the F 1s peak is significantly broader, possibly consistent with the presence of more than one unresolved component. The poorer statistics of the N 1s spectrum are due to its low photoionization cross section and the high background of the inelastic ‘tail’ of the intense Au 4d emission; longer data collection times were avoided to minimize radiation damage.

NIXSW measurements employing the Au(111) reflection led to the determination of values of the two key associated structural parameters, the coherent fraction, *f*, and the coherent position, *p*. The coherent fraction is commonly regarded as an order parameter, while the coherent position is the offset of the absorber position, in units of the substrate layer spacing *d*_(111)_, relative to the nearest extended substrate (111) plane. This can be related to the true height relative to this plane, *D* = (*p* + *n*) *d*_(111)_, where *n* is an integer chosen to ensure that interatomic distances are physically reasonable.^6^ There is very rarely any ambiguity in this choice. The resulting values of *f* and *D* for each chemically distinct absorber atom are shown in Table 1. The experimental absorption profiles and the fits based on these values of the structural parameters are shown in Figure S1 of the Supporting Information.

**Table 1:** Summary of the Coherent Fractions, *f*, and Coherent Position Values (the Latter Converted into a Physical Distance, *D*) Obtained from the NIXSW Measurements^a^

	*f*	*D* (Å)
CF	0.70(10)	3.29(15)
CC	0.66(10)	3.27(15)
CN	0.40(10)	3.22(20)
N	0.10(10)	2.82(40)
F	0.37(10)	3.52(20)

a Estimated precisions are shown in parentheses in units of the least significant figure. Precision estimates for *D* in the cases of very low values of *f* take account of the problems of achieving meaningful values of *D* discussed elsewhere.^23^

As remarked above, the coherent fraction is generally regarded as an order parameter; if all absorber atoms are at the same height relative to the (111) planes, with no static or dynamic disorder, the value of *f* would be unity. Co-occupation of two or more different heights can lead to much lower values, but even in the case of only a single height being occupied, thermal vibrations of the substrate atoms (which introduce an incoherent scattering background to the standing wave), and of the absorber atoms within the standing wave, must reduce this value by appropriate Debye–Waller factors. Careful evaluation of the possible type of disorder that can occur in adsorbed molecular layers^30^ leads to the conclusion that values of less than ∼0.75 are likely to indicate contributions from coexisting different heights of the corresponding atomic species. The values of 0.70 for the CF atoms and 0.66 for the CC atoms fall slightly outside this limit but may be large enough to suggest that the great majority of molecules do have these C atoms at single well-defined heights. By contrast, the value for the N atoms is extremely low, clearly indicating co-occupation of at least two distinctly different heights. The situation is similar, albeit less extreme, for the CN and F atoms. Precision estimates for the *D* values for these atoms include allowance for the large uncertainty arising from the low values of the coherent fractions.^30^ In this context, we note that two different preparations of the adsorbate surface, both yielding the same LEED pattern but with very different average coverages of F_4_TCNQ as determined by XPS, yielded significantly different NIXSW coherent fractions values. Specifically, the higher coverage preparation showed low coherent fractions in the range 0.3–0.5 for all of the absorber atoms; as argued elsewhere,^30^ this effect can only easily be reconciled with at least partial double-layer or multilayer growth of the molecular overlayer. The lower coverage data are therefore expected to be a much better representation of the ordered single-layer phase of interest, although it is possible that even in this case the slightly reduced *f* values for the CC and CF species may be due to a small fraction of the molecules occupying a second (or higher) layer.

The very much lower value of *f* for the N atoms, however, cannot be accounted for in this way; this must imply that the N atoms occupy at least two distinctly different heights above the surface, differing by up to 1 Å, in the ordered monolayer. Exactly this effect was seen in an investigation of the commensurate ordered phase of TCNQ on Ag(111)^7^ and was shown to be reconcilable with a twisted molecular conformation, attributed to the presence of Ag adatoms. Specifically, the four N atoms of each molecule adsorb at two different heights, leading to the twisted molecular conformation, the upper N atoms being bonded to the adatoms, while the lower N atoms are bonded to the undisturbed underlying Ag surface atoms. It therefore seems likely that a similar geometry occurs in the Au(111)-F_4_TCNQ system if Au adatoms are involved. Notice that if N atoms occupy two or more different heights, this is also likely to occur for the CN atoms bonded to the N atoms, leading to a significant reduction in *f* for these absorbers.

The low coherent fraction of the F atoms also indicates the co-occupation of multiple heights. Higher-resolution XP F 1s spectra recorded from F_4_TCNQ adsorbed on Ag(100)^31^ show the presence of a second component, weakly resolved at the higher energies of the NIXSW measurements, that appears to be related to radiation damage. This suggests that on both of these surfaces the F NIXSW results may be influenced by the presence of a coadsorbed atomic F species. In this context, we note that F is known to have a particularly high cross section for electron- and photon-stimulated desorption (*e.g*.^32^).

### Density Functional Theory Calculations

As remarked in the introduction, the earlier STM-based investigation of the Au(111)-F_4_TCNQ system^9^ did report results of DFT calculations for an adsorption-induced adatom structure, but the focus of these calculations was primarily on simulating STM images. No quantitative structural parameters were reported. However, a schematic side view of the adsorption structure included in this paper appears to show the molecule in the inverted bowl conformation commonly reported in DFT calculations of TCNQ adsorption on metal surfaces without adatoms being present. For the present quantitative structure determination, a comparison of the calculated structural parameters with the results of the experimental NIXSW results is essential, and for this purpose, the inclusion of dispersion forces is now widely recognized as being very important. We also wish, within this work, to establish from DFT calculations the energetic advantage of Au adatom incorporation into the F_4_TCNQ overlayer, a quantity not explicitly reported in earlier studies of this system.

Our DFT calculations were performed for two alternative structural models of the  ordered phase, one in which there is only a single F_4_TCNQ molecule in each unit mesh, the other in which each unit mesh contains one F_4_TCNQ molecule and one Au adatom. The minimum-energy version of each structure was then analyzed to extract values for the NIXSW structural parameters that would be expected from these structures. Notice that the DFT values of the coherent fractions only take account of the reduction from the ideal value of unity due to height variations of chemically equivalent atoms in the overlayer. No account is taken of static or dynamic disorder, so the theoretical values of the coherent fractions may be expected to be up to 30% too large for realistic comparison with experimental values.^23^ Notice, too, that if an atomic species occupies two different heights that differ by one-half of the bulk interlayer spacing the coherent fraction falls to zero, so the value of the coherent fraction is extremely sensitive to the height difference as it approaches this value.^30^

**Figure 3:**
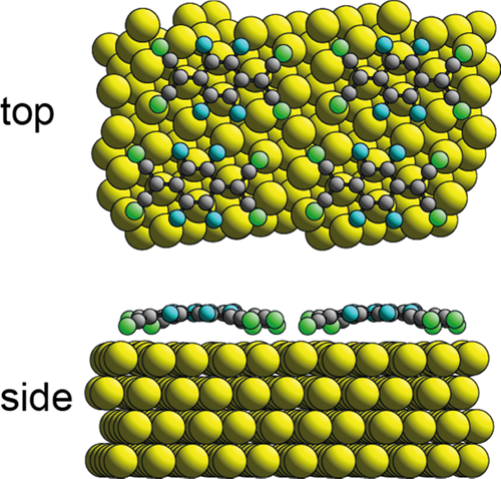
Top and side views of the minimum-energy DFT structure of the model based on F_4_TCNQ adsorbed on Au(111) in a  unit mesh without Au adatoms. Au atoms are shown colored yellow, C black, F blue, and N green.

Table 2 shows this comparison of experimental and theoretical NIXSW parameter values. For the no-adatom model, the predicted heights of the C atoms in and close to the central quinoid ring are in good agreement with the experimental values, although the agreement with the experiment for the heights of the other atoms is relatively poor. Most significantly, however, the no-adatom model predicts all of the chemically equivalent atoms to be at closely similar heights above the surface, resulting in high predicted values for their coherent fractions. In particular, the model completely fails to account for the exceptionally low value of the coherent fraction for the N atoms. As shown in Figure 3, in the no-adatom model, the molecule adopts an inverted bowl configuration with all N atoms, at clearly similar heights, approximately 0.5 Å lower on the surface than the central quinoid ring. This molecular conformation was also found in previous DFT calculations of F_4_TCNQ^10,11^ (and also TCNQ^16^) adsorbed on an unreconstructed Au(111) surface at low coverage (without the constraint of the dense packing of the low-symmetry  unit mesh).

**Table 2:** Comparison of the Experimental NIXSW Parameter Values of Table 1 with Predicted Values for the Optimized DFT Structures of Two Alternative Models, with and without Au Adatoms^a^

	expt.	DFT with adatom	DFT no-adatom	expt.	DFT with adatom	DFT no-adatom
	*f*	*f*	*f*	*D* (Å)	*D* (Å)	*D* (Å)
CF	0.70(10)	1.00	1.00	3.29(15)	3.25	3.28
CC	0.66(10)	0.98	0.98	3.27(15)	3.23	3.15
CN	0.40(10)	0.70	0.95	3.22(20)	3.03	2.86
N	0.10(10)	0.37	0.82	2.82(40)	2.83	2.62
F	0.37(10)	0.98	1.00	3.52(20)	3.23	3.31

a DFT atomic heights are relative to the average outermost Au layer.

**Figure 4:**
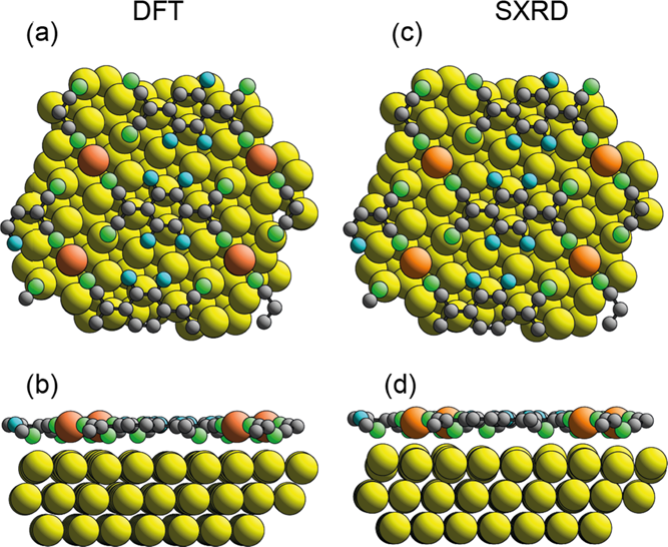
Top (a) and side (b) views of the minimum-energy structure (including Au adatoms) of the  Au(111)-F_4_TCNQ phase found in the DFT calculations with a superimposed unit mesh. The Au adatoms are shaded orange to distinguish them from those of the unreconstructed substrate: C atoms are shown gray, N green, and fluorine blue. (c, d) Structure found in the SXRD study.

By contrast, the structural model including one Au adatom per surface unit mesh predicts a very low experimental coherent fraction for the N atoms. This arises because in the adatom structural model (Figure 4a,b), the F_4_TCNQ molecule has twisted ends, leading to two very different heights (by ∼0.8 Å) of the N atoms (and two slightly less different heights of the C–N atoms), causing a significant reduction of the associated coherent fractions, consistent with the experimental data. The exceptionally low experimental coherent fraction for the N atoms, in particular, leads to very poor precision in the coherent position, expressed as a value of *D*. With this caveat, the agreement between the experimental *D* values for all chemically distinct atoms and those predicted for the adatom models is good.

Based on the combination of NIXSW and DFT structural data, there is therefore clear indirect evidence of the presence of adatoms in the Au(111)-F_4_TCNQ  structure. A theoretical analysis of the energetics of the two models reinforces this conclusion. Adsorption energies (*E*_ads_) per surface unit mesh (and thus per F_4_TCNQ molecule) were calculated for the no-adatom model (see Figure 3) as
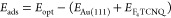
1where *E*_opt_, *E*_Au(111)_, and *E*_F_4_TCNQ_ are the total energy of the optimized structure, of the clean Au(111) (unreconstructed) surface, and of the free molecule, respectively. For the adatom model (Figure 4a,b), eq 1 is modified to
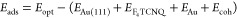
2where *E*_Au_ is the energy of a free Au atom (included to account for the additional Au atom per unit mesh in the adatom structure) and *E*_coh_ is the cohesive energy of bulk Au (included to account for the energy cost of extracting the adatom). In these formulations, all energies are taken to be positive. The results reveal a strong energetic advantage for the adatom model with an adsorption energy per unit surface area of 3.35 eV/nm^2^ relative to a value of 2.57 eV/nm^2^ in the absence of the adatom. This situation is similar to that of F_4_TCNQ on Ag(100)^31^ for which we showed that the strong intralayer bonding in the 2D-MOF that is formed is the origin of the preference for the adatom model. The results show the optimum lateral registry of the overlayer to the underlying Au(111) surface to correspond to the Au adatoms occupying local atop sites; shifting this registry to a hollow site reduced the adsorption energy by 0.43 eV/nm^2^, while starting in a bridge site, the calculation converged on the hollow site geometry. Figure 4a,b shows the minimum-energy structure (which includes Au adatoms) in both plan and side views. A comparison with the results of the earlier DFT study of the adatom structure is not really possible because the equivalent diagram shown in Figure 1 of this earlier paper^9^ can only be regarded as highly schematic, showing a model with a completely different  unit mesh having an area 3 times larger than the true  unit mesh, with the superimposed F_4_TCNQ molecule apparently being enlarged to fit this large mesh.

A comparison of the NIXSW and DFT structural results, and the DFT energetics, clearly favors the adatom model. However, these techniques do not provide any direct experimental evidence of the presence and location of Au adatoms. This information is provided in the results of the SXRD experiment described below.

### SXRD

SXRD measurements focused predominantly on the intensities of the fractional order diffracted beams arising from the  unit mesh, with beams corresponding to a single rotational and mirror reflection domain of the complete diffraction pattern being selected. SXRD data collection generally involves three types of measurements, namely, (i) the intensities of fractional order diffracted beams,  at a low value of , referred to as ‘in-plane’ intensities ( being the component of momentum transfer perpendicular to the surface); (ii) ‘rod scans’ of the intensities of these fractional order beams as a function of  (known as ‘fractional order rod (FOR) scans’); and (iii) rod scans of integer order beams, known as crystal truncation rod (CTR) scans. The data in (i) and (ii) are influenced only by the structure of that part of the surface that shows the periodicity of the surface phase, providing no information on the location of these atoms relative to the unreconstructed substrate, and (iii) contains information on the complete structure including that of the substrate. The dataset collected from the Au(111)-F_4_TCNQ system, identified in Figure S2, comprised mainly in-plane intensities (i) but also included a small number of FORs and CTRs, which were restricted in their range of  by the low photon energy (large wavelength) chosen to avoid a strong background signal of Au fluorescent X-rays.

**Figure 5:**
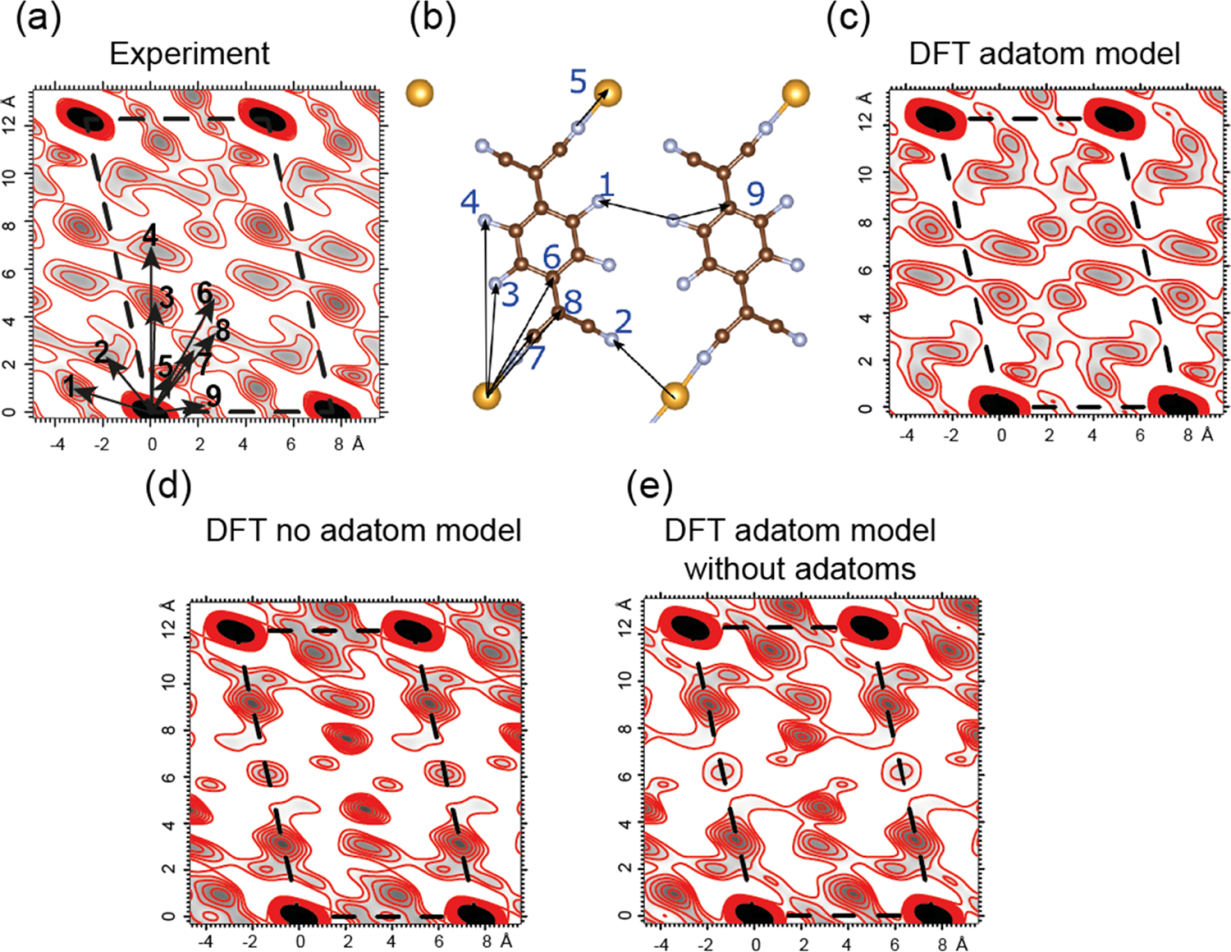
(a) Patterson map obtained from the experimental in-plane fractional order diffracted beam intensities with dominant numbered Au–F, Au–N, and Au–C interatomic vectors superimposed. (b) Interatomic vectors shown in (a) correspond to the real-space structure. Some overlapping intramolecular vectors are shown in Figure S3. (c) Patterson map produced from calculated intensities from the adatom structure found in the DFT calculations. Patterson maps based on calculated intensities from the optimal DFT structure for a no-adatom structure, and for the adatom structure with the adatoms removed, are shown in (d, e).

While a full structure determination based on these data requires computer simulations derived from alternative model structures, a Patterson function map of the projection of the structure onto the surface plane can readily be produced directly from the set of in-plane measurements of the fractional order beams (*e.g*.^33^). A Patterson function map (essentially a Fourier transform of the diffracted intensities) does not show the spatial variation of the electron density directly due to the loss of phase information in these intensities (as opposed to the amplitudes), but does show a self-convolution of this quantity, and is thus a map of interatomic vectors.^34^ The Patterson map obtained directly from the experimental data is shown in Figure 5a, with the unit mesh superimposed, together with some of the dominant interatomic vectors labeled. The map is clearly dominated by one intense peak at each corner of the unit mesh. These arise from all vectors from atoms within one unit mesh to the equivalent atom in an adjacent unit mesh (including Au–Au vectors); these correspond to the primitive translation vectors of the surface mesh, so these dominant peaks show only the periodicity of the surface. However, significant structural information is provided by the features of the Patterson map within the unit mesh. These are expected to be dominated by Au–C, Au–F, and Au–N vectors; intramolecular (C–C, C–F, C–N) vectors are expected to be weaker due to the smaller scattering cross sections for these atoms, although multiple similar interatomic vectors present within the molecule may also make some of these features visible in the map. Figure 5b shows the main interatomic vectors expected to dominate the Patterson map for the Au adatom structure, which clearly do correspond to the main features of the experimental map. This correspondence is reinforced by Figure 5c, which shows that the Patterson map generated from calculated fractional order beam intensities for the lowest-energy adatom structural model is found in the DFT calculations (Figure 4a). This is clearly almost identical to the Patterson map of the experimental data in Figure 5a. By contrast, a Patterson map generated from calculated fractional order beam intensities for the same (adatom) structural model favored by the DFT calculations, but omitting the Au adatoms, Figure 5e, and for the lowest-energy DFT no-adatom structure (Figure 5d) is very different. This provides clear evidence that the features of the experimental Patterson map within the unit mesh are consistent with the Au–C, Au–N, and Au–F vectors expected for the adatom structure. Thus, these results constitute a clear demonstration of the initial objective of this investigation: to show that SXRD does provide a way to ‘see’ metal adatoms in an unambiguous fashion. Moreover, there is a clear qualitative agreement of the structure between the results of the DFT calculations and the SXRD experiments.

A full quantitative structure determination by SXRD is achieved by the trial-and-error approach common to almost all surface structural techniques, in which the measured quantities, in this case diffracted beam intensities, are compared with computed values to be expected for different structural models. For SXRD, these computed values are provided using the ROD computer program.^35^ In the present case, there is potentially a very large number of structural parameters to be determined. Specifically, these include the three Cartesian coordinates of each of the 24 constituent atoms of the F_4_TCNQ molecule and of the Au adatoms but also the heights above the substrate of the 13 Au atoms per surface unit mesh in each of the outermost Au(111) layers. These layers may be rumpled as a consequence of the molecular bonding (significant rumpling in the outermost two layers is predicted by the DFT calculations). Even with our dataset comprising 82 in-plane fractional order beam intensities, 3 FORs, and 3 CTRs, optimizing all of these parameter values independently is an unrealistic goal.

Unsurprisingly, exhaustive searches of models in which the coordinates of the weakly scattering C, N, and F atoms within the adsorbed molecule were varied, led to the conclusion that SXRD is too weakly dependent on these parameters to reach any conclusions about the exact molecular conformation. The small improvements in the quality of fit to the experimental data that were found often involved unphysical changes in intramolecular bond lengths and bond angles.

The details of the subsequent strategy for structural optimization used in the trial-and-error modeling are described in the Supporting Information. This was conducted using the complete experimental dataset of in-plane and out-of-plane diffracted beam intensities identified in Figure S2. Briefly, these calculations assumed that the molecular conformation was the same as that found in the DFT calculations. The remaining parameters to optimize were therefore the relative heights and lateral registry of the adsorbed molecule and the Au adatoms but also the layer spacings and rumpling of the outermost Au surface layers; this rumpling proved to be important to achieve the best agreement between theory and experiment.

**Figure 6:**
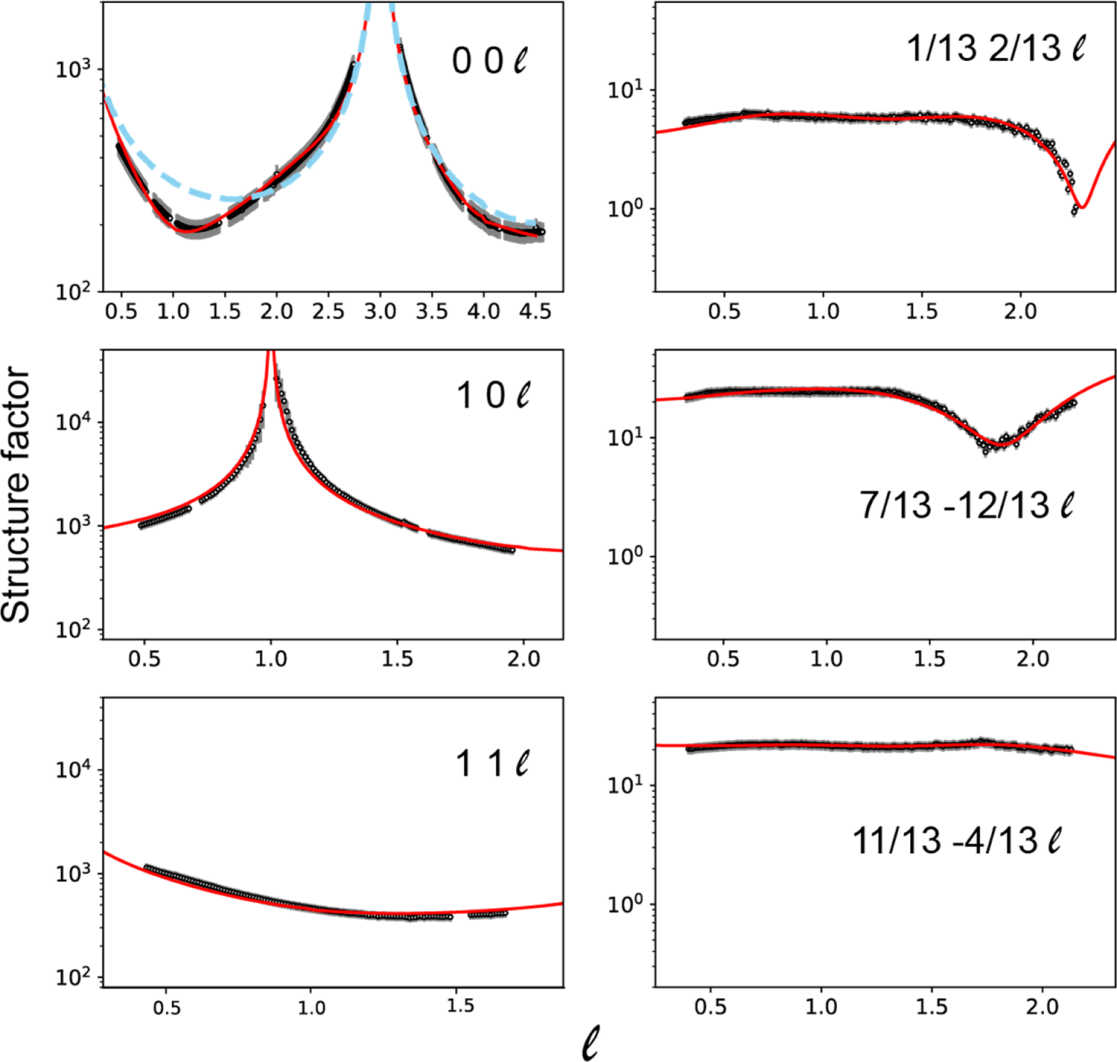
Experimental rod scans (individual data points shown in black with error bars) compared to the results of the ROD simulation for the optimized structural model (red continuous lines). The blue dashed curve in the 0 0  panel corresponds to the results of a calculation from a clean unrelaxed Au(111) surface.

Figure 6 shows a comparison of the experimental rod scans to the results of calculations for the best-fit structure, while the comparison of the experimental and computed in-plane data is shown in Figure S4. The agreement is clearly good. Table 3 summarizes the overlayer spacing found in the SXRD analysis, compared to the equivalent values from the DFT calculations and the NIXSW experimental results. Note that as the molecular conformation in the SXRD calculations was assumed to be that of the DFT calculations, reporting separate values for the heights of the constituent atoms in this comparison is not really meaningful, so only the heights of the central quinoid ring are compared in this table. The SXRD precision estimates for the molecule and adatom heights are determined by the range of individual parameter values that lead to a value of chi-squared within 5% of the best-fit value; there was some evidence of coupling between the values of the adatom height and the rumpling amplitudes, so the error estimates taking account of this may be increased to about ±0.10 Å. Clearly, all three methods agree within these estimated experimental precisions. A key conclusion is therefore not only that the SXRD results show that the Au adatoms do exist in the overlayer but also that their height above the surface is determined to be consistent with the DFT model. In detail, the Au adatoms in the SXRD structure are 0.27 ± 0.06 Å below the molecular layer, compared to 0.14 Å in the DFT model. One further important finding, however, is that the SXRD fit shows that the amplitude of the rumpling of the outermost Au layer, in particular, is significantly larger than that indicated by the DFT calculations. Specifically, the SXRD results show that the rumpling amplitudes of the outermost and second Au(111) layers are 0.60 ± 0.06 and 0.18 ± 0.07 Å, respectively, when compared to values of 0.20 and 0.09 Å, respectively, in the DFT model. The rumpling amplitude is defined here as the difference in height of the highest and lowest atoms within the layer, but the particularly large value of this amplitude found in the SXRD analysis for the outermost layer is attributable to the two Au atoms that lie directly below the N atoms that are not bonded to Au adatoms, which lie 0.60 Å above the lowest top layer atoms. As these Au atoms are forming Au–N bonds to the molecule, a larger outward displacement of these atoms is qualitatively reasonable, but the large magnitude of this effect is difficult to reconcile with a shift of these atoms of only 0.1 Å in the DFT results. The rumpling of the remainder of the outermost layer atoms in the SXRD model is 0.38 Å.

**Table 3:** Comparison of the Height of the Central Quinoid Ring, and of the Au Adatom, above the Average Outermost Au Layer Obtained from NIXSW, SXRD, and the DFT Calculations for the Adatom Model of the  Au(111)-F_4_TCNQ Phase

atom	DFT (with adatom) *D* (Å)	NIXSW *D* (Å)	SXRD *D* (Å)
C quinoid	3.24	3.29 ± 0.15	3.36 ± 0.05
Au adatom	3.10		3.09 ± 0.04

The fact that the SXRD analysis indicates significantly enhanced surface layer rumpling, relative to that predicted by the DFT calculations, highlights another potentially important feature of the SXRD technique. Experimental data on this rumpling effect are rather scarce because most surface structural techniques used to investigate molecular adsorption structures focus on the relative location of atoms in the adsorbate and are ‘blind’ to displacements of the substrate atoms. The notable exception is quantitative LEED (QLEED), also known as LEED I–V (intensity–voltage) analysis, which is closely similar to SXRD but with X-rays replaced by low-energy electrons. This technique^36^ has identified substrate surface rumpling in atomic adsorption and adsorption of diatomic molecules such as CO but is extremely challenging to apply to large surface mesh structures associated with adsorption of larger molecules because the importance of multiple scattering leads to much higher computational demands than SXRD. The origin of the difference in rumpling amplitude between the SXRD structure and the DFT result is unclear but may be related to the limited number of Au atomic layers in the DFT ‘slab’, due to the computational demands of this large surface mesh structure. However, it would be surprising if this could account for such a large difference in the rumpling amplitude of the outermost layer.

Fits to the SXRD data with the lateral registry of the Au adatoms constrained to the four alternative high-symmetry sites of the outermost Au layer clearly support the same atop site as the DFT calculations. The lowest chi-squared value, 1.236, was found for this atop site, whereas the values for the alternative registries were in the range of 1.35–1.69. Table S1 shows the specific values and structural parameter values for each alternative registry. Notice that the Au adatom–Au surface atom nearest neighbor distance in this atop registry is 2.99 Å according to the DFT model, which is significantly larger than the Au–Au spacing in the bulk crystal (2.88 Å), suggesting that there is no Au adatom–Au surface atom bonding. This would imply that the height of the Au adatom above the surface is determined more by the molecule–substrate and molecule–adatom bonding than by any adatom–substrate bonding. This behavior would be similar to that seen in the two-dimensional metal–organic framework formed by K coordinated to TCNQ on the Ag(111) surface, in which the K atoms occupy off-atop sites.^37^ The value of this Au–Au adatom spacing obtained from the SXRD analysis of 3.06 ± 0.06 Å is even larger. As a more quantitative test of the sensitivity of the SXRD data to the presence of the Au adatoms, further ROD calculations were performed starting from the DFT no-adatom model and using the same strategy as for the adatom model to search for modifications of this structure that yielded the best fit to the experimental data. The chi-squared value for the resulting model was 6.611, to be compared with the value of 1.236 for the adatom model. This model gave a particularly poor fit to the in-plane fractional order intensities as shown in Figure S6, with almost all predicted structure values being significantly too low, consistent with the absence of the strongly scattering Au adatoms.

## Conclusions

Despite several reports of indirect evidence of molecular adsorption on noble metal surfaces leading to the presence of metal adatoms that are incorporated into the molecular overlayer, there has been no positive experimental identification by a quantitative structural technique. The main evidence has come from interpretation of STM images that, even aided by simulated images based on DFT calculations and the use of the Tersoff–Hamman approach, are not unambiguous. By contrast, X-ray diffraction is an extremely well-established technique, the theory of which is well understood and has been used to solve crystal structures of great complexity, notably in macromolecular chemistry and the life sciences. Here, we have shown that surface X-ray diffraction can be used to provide the necessary unambiguous identification of Au adatom creation following the adsorption of F_4_TCNQ on Au(111). While this adsorption system was identified as a likely example of this adatom incorporation by an earlier combined STM/DFT study, our new study has clarified many important aspects of this model system, including (i) experimental determination of the height of the adsorbed molecule above the surface using NIXSW; (ii) evidence from NIXSW experimental data that the adsorbed molecule is twisted and does not adopt an inverted bowl configuration as previously proposed; (iii) application of dispersion-corrected DFT calculations to determine the preferred adsorption geometry, which is in excellent agreement with the experimental NIXSW results and the conclusions regarding the molecular conformation; (iv) use of dispersion-corrected DFT calculations to quantify the strong energetic advantage of the adatom incorporation structure over adsorption on an unreconstructed surface; (v) demonstration that experimental surface X-ray diffraction unambiguously shows the presence of Au adatoms in the adsorption structure, with the molecule and adatom heights above the surface, and the lateral registry of the overlayer, consistent with the DFT results; and (vi) the importance of rumpling in the outer Au layers.

Clearly, these results show that SXRD can be used to explore the phenomenon of adsorption-induced adatom creation in a range of other systems and has proved to be a crucial complementary experimental technique to achieve a better understanding of this effect.

## Supporting Information Available

NIXSW results; SXRD structure analysis details (PDF)
