# Supplementary material for: Direct Experimental Evidence for Substrate Adatom Incorporation into a Molecular Overlayer
Source: J Phys Chem C Nanomater Interfaces. 2022 Apr 19;126(16):7346–55. doi: 10.1021/acs.jpcc.2c01432 (PMC9059187; doi:10.1021/acs.jpcc.2c01432)
Supplement: Supplementary file 1 — jp2c01432_si_001.pdf [file jp2c01432_si_001.pdf]

Supporting Information

Direct Experimental Evidence for Substrate Adatom Incorporation into a Molecular Overlayer

Philip J. Mousley[1], Luke A. Rochford[2], Paul T.P. Ryan[1,3], Philip Blowey[1,4], James Lawrence[5], David A. Duncan[1], Hadeel Hussain[1], Billal Sohail[5], Tien-Lin Lee[1], Gavin R. Bell[4], Giovanni Costantini[5], Reinhard J. Maurer[5], Christopher Nicklin[1], D. Phil Woodruff[4*]

[1]*Diamond Light Source, Harwell Science and Innovation Campus, Didcot, OX11 0DE,*

[2]*Chemistry Department, University of Birmingham, University Road, Birmingham B15 2TT, UK*

[3]*Department of Materials, Imperial College, London SW7 2AZ, UK*

[4]*Department of Physics, University of Warwick, Coventry CV4 7AL, UK*

[5]*Department of Chemistry, University of Warwick, Coventry CV4 7AL, UK*

1. **NIXSW results**

2. **SXRD structure analysis details**

1. NIXSW results

Figure S1 shows a comparison of the raw NIXSW photoemission intensity scans with the best-fit theoretical curves, the two fitting parameters (coherent fraction and coherent position) being reported in Table 1 of the main paper. Values of the backward-forward asymmetry factor, *Q*, were 0.11 for the C and N emitters and 0.10 for the F emitter.

* Email: d.p.woodruff@warwick.ac.uk

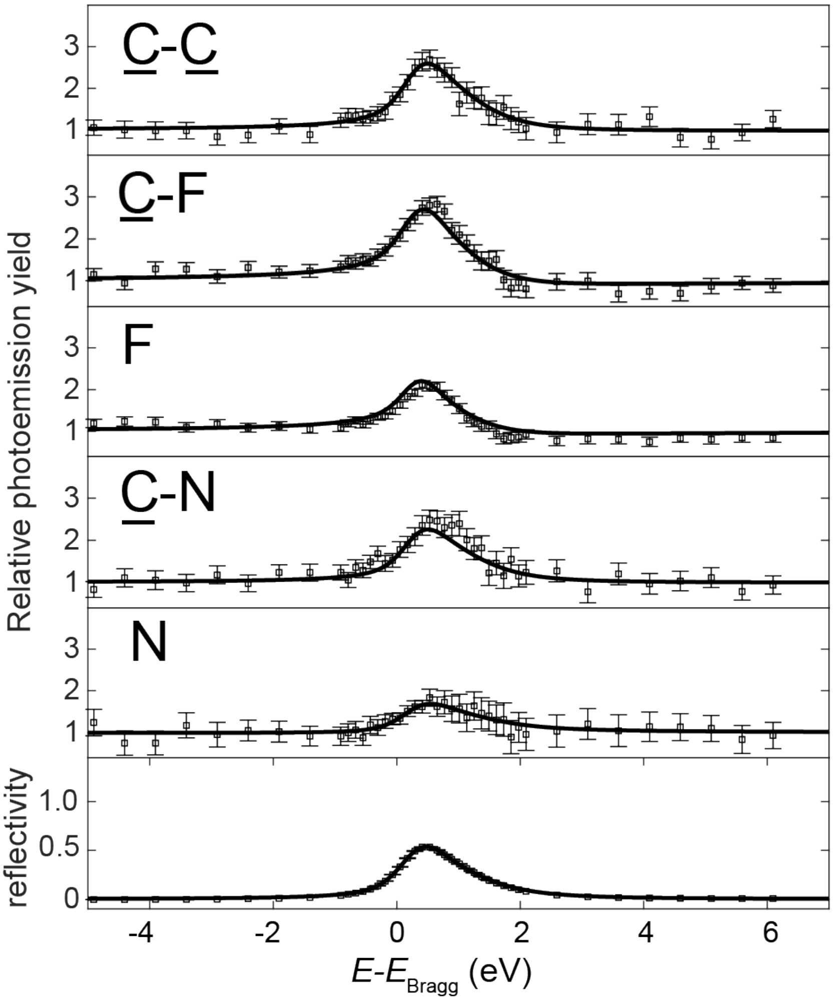

Figure S1 Individual experimental chemical-state-specific NIXSW scans (data points) with the superimposed fits corresponding to the values of the coherent fractions and position reported in Table 1 of the main paper.

2. SXRD structure analysis details

The complete structural data set measured from a single rotational/mirror domain of the Au(111)-F_4TCNQ adsorption phase comprised 82 fractional-order beam 'in plane' intensities, three CTR scans ((00ℓ), (10 ℓ) and (11ℓ)) and three FOR scans ((1/13, 2/13 ℓ), (7/13 -12/13 ℓ) and (11/13 -4/13 ℓ)), this labelling being based on a $\begin{pmatrix} 2 & -1 \\ 3 & 5 \end{pmatrix}$ matrix; Figure S2 specifies these.

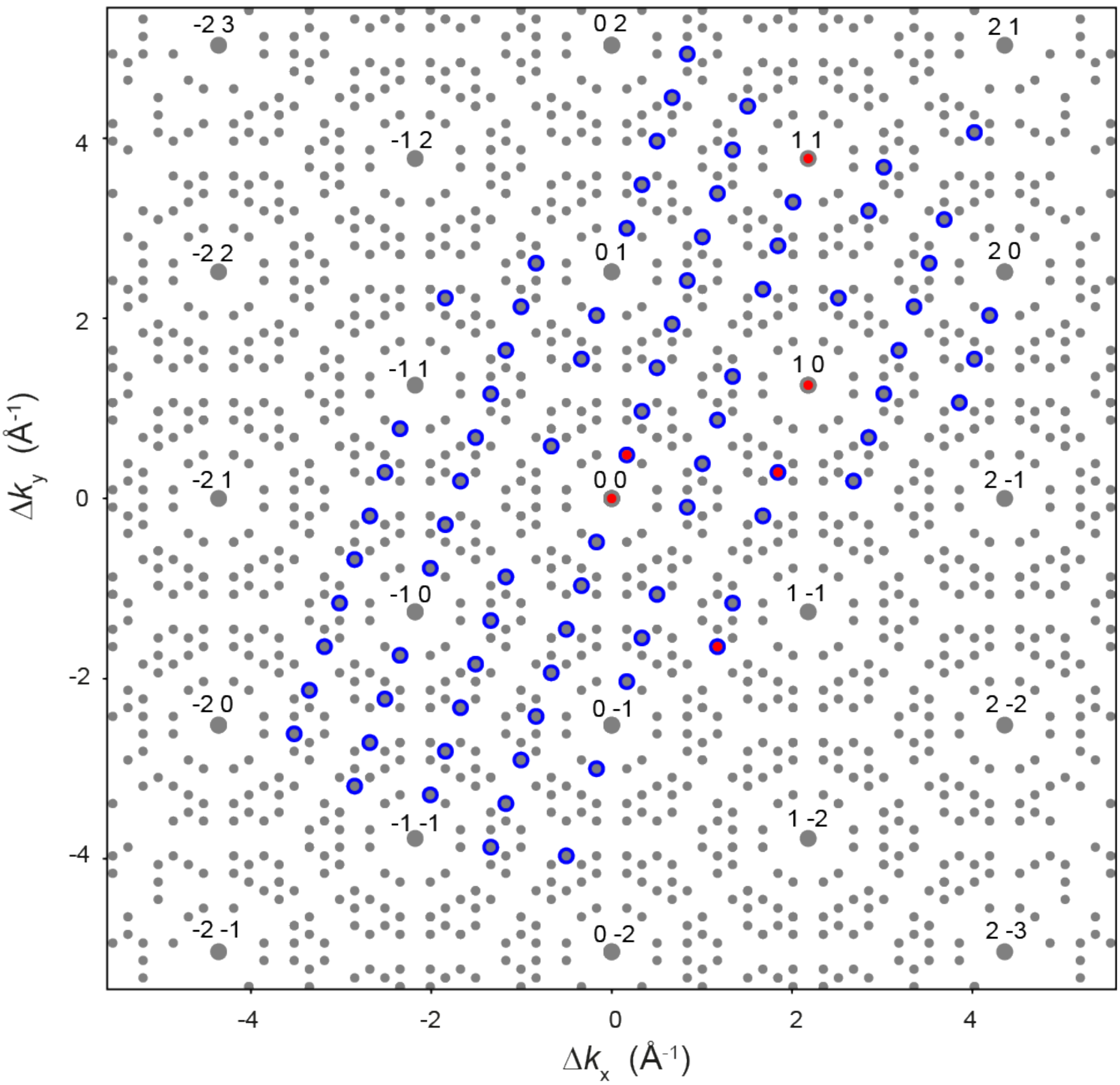

Figure S2 Representation of the diffraction pattern of the Au(111)-F_4TCNQ adsorption phase from all coexistent rotationally and mirror reflected domains, identifying the SXRD dataset measured from a single such domain of the complete pattern. In-plane intensities were measured from the beams circled in blue, while rod scans were obtained from those marked with red circles. Integral order beams are labelled.

In principle, 'in plane' intensities correspond to (hk0) beams – i.e. to measurements with the perpendicular momentum transfer ℓ equal to zero. In reality, measurements are not possible at ℓ =0, but can be obtained at small values of ℓ. In the present case these 'in plane' intensities

were measured at $\ell = 0.2$. Friedel's rule tells us that the intensity of the $(-h-k\ell)$ beam is the same as an $(hk-\ell)$ beam, so an average of the intensities of an $(-h-k\ell)$ beam and the corresponding $(hk\ell)$ beam at low values of ℓ can provide a good interpolated estimate of the true in-plane $(hk0)$ intensity. However, for the dataset collected, calculations of the ℓ dependence of the fractional order beam intensities based on the DFT structrural model indicated that this linear interpolation approach could lead to intensity errors of ~10% or more in some cases, so all theory-experiment calculations were based on the true measured values of ℓ, based on a symmetry of P1; thus, no symmetry averaging was applied.

The Patterson map based on these in-plane measurements is shown in Figure 5 of the main paper; included in this figure as part 5(b) is a real-space representation of the structure showing some of the dominant interactomic vectors, mostly involving Au adatoms. Figure S3 shows an extended set of interatomic vectors superimposed on a model of the real space structure covering 4 unit meshes, also including some multiply-occuring inter- and intra-molecular vectors.

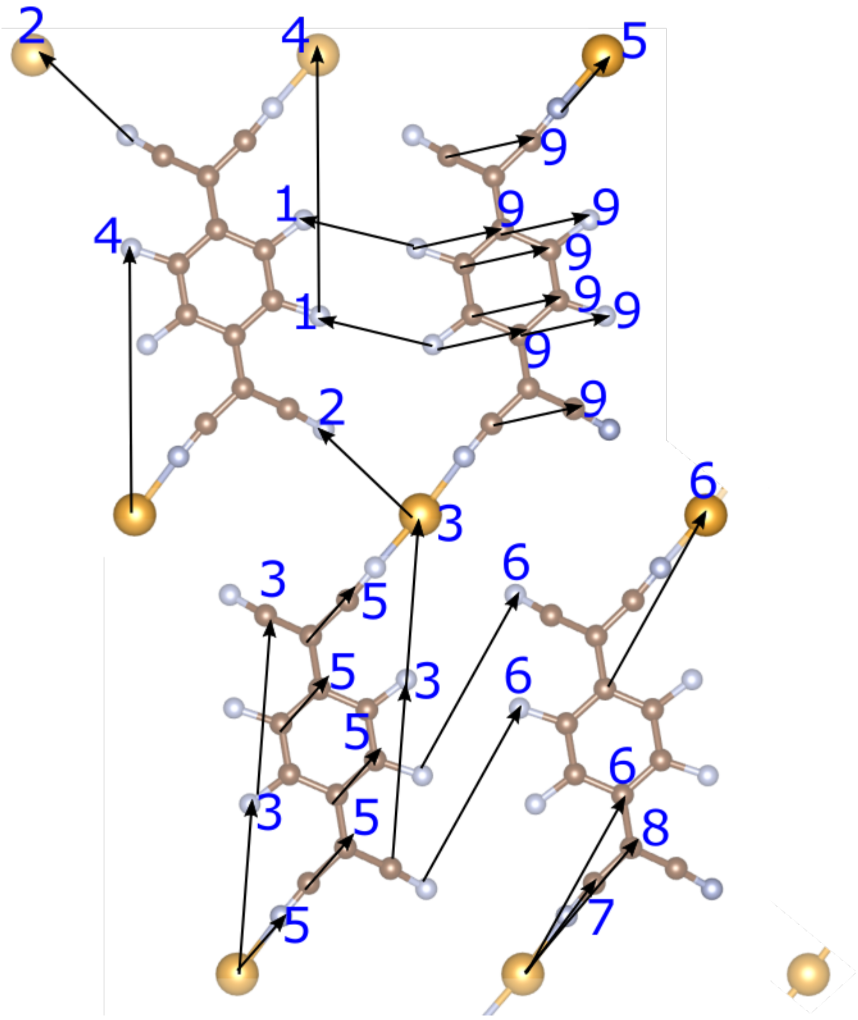

Figure S3 Diagram of the surface structure over four unit meshes showing the repeat occurrences of in-plane vectors labelled 1-9, identified in the Patterson map shown in Figure 5(a) of the main paper. The number at the head of each arrow corresponds to its vector label.

Full quantitative structure determination using SXRD relies on the trial-and-error fitting process common to almost all surface structural technques, the experimental diffracted beam intensities being compared with those predicted in simulations based on a series of alternative structural models. For this purpose, calculations used the ROD computer program.[1] Initially the parameter search was performed on models based on a planar F_4TCNQ molecule together with an Au adatom placed at different heights and lateral positions on a bulk-terminated substrate, adjusting a range of parameters defining distortions of the molecular conformation including in-plane and out-of-plane displacements of individual atoms. The experimental dataset used for this initial theory/experiment comparisons was the complete set of 'in plane' fractional-order beam intensities and the rod scan of just one fractional order beam, namely (1/13 2/13 ℓ). The main conclusion of exhaustive structural searches based on this approach was that the experiental data were too insensitive to detailed parameters defining the molecular conformation to reach any conclusions on this aspect of the structure. This conclusion is, of course, consistent with the known weak scattering cross-sections of the atomic constituents of

the molecule. Further structural searches therefore assumed the molecular conformation was as given by the DFT calculations; this choice was reinforced by the fact that the relative heights of the constituents atoms in this model are consistent with the NIXSW experimental results.

A second stage of the structure determination focussed on the (00) CTR with the objective of identifying the various layer spacings (of the molecule and adatom, but also of any relaxation of the outermost substrate layer spacings), an approach based on the recognition that this specular reflectivity scan is sensitive *only* to these layer spacings. These calculations showed that the fit to the (00) rod is extremely sensitive to the spacings of the outermost substrate layers (with changes of only 0.01 Å leading to detectable changes), but far less sensitive to the heights of the molecule and Au adatom, changes of up to ~0 2 Å in these parameters leading to changes within the precision limits of the expetimental measurements. This difference in sensitivity reflects the difference in scattering cross-sections of complete Au(111) layers (each containing 13 Au atoms per surface unit mesh) and layers containing only one Au adatom or one complete molecule (comprising low atomic number atoms). Despite the relatively weak sensitivity to the exact location of these layers, a good fit to the data could only be achieved with both the molecule and the adatom being present in the structural model. The (00) CTR also proved to be sensitive to changes in the rumpling amplitude of the outermost most layers.

In the third stage of the structural optimisation, the dataset used for comparison was enlarged to include the full set of three fractional order rod scans and three integral order CTRs (see Figure S3), as well as the complete in-plane dataset. This revealed a strong sensitivity, particularly for the fractional order rods, to the surface layer rumpling amplitudes. Modifying the specifics of the DFT rumpling (i.e. which surface atoms within the surface unit mesh were displaced by how much), or refining the details of rumpling added to a model with initially planar surface layers, did not significantly alter the quality of the experiment-theory fits, but variations in the amplitude of the rumpling did prove to be important. Initially, the effect of modifying the details of the rumpling of the DFT model was explored. the final oprimsed structure was obtained by starting with planar outer layers and allowing the full range of displacements perpendicular to the surface of all the independent Au atoms in the two outermost layers.

The optimised surface structure obtained using this approach, which yielded a chi-squared value of 1.236, is illustrated in Figures 4(c) and (d) of the main paper, with the fits to the rod

scans shown in Figure 6, while the fit to the in-plane data for this model is shown in Figure S4. The adatom and molecule layer spacings for this structure are listed in Table 3 of the main paper.

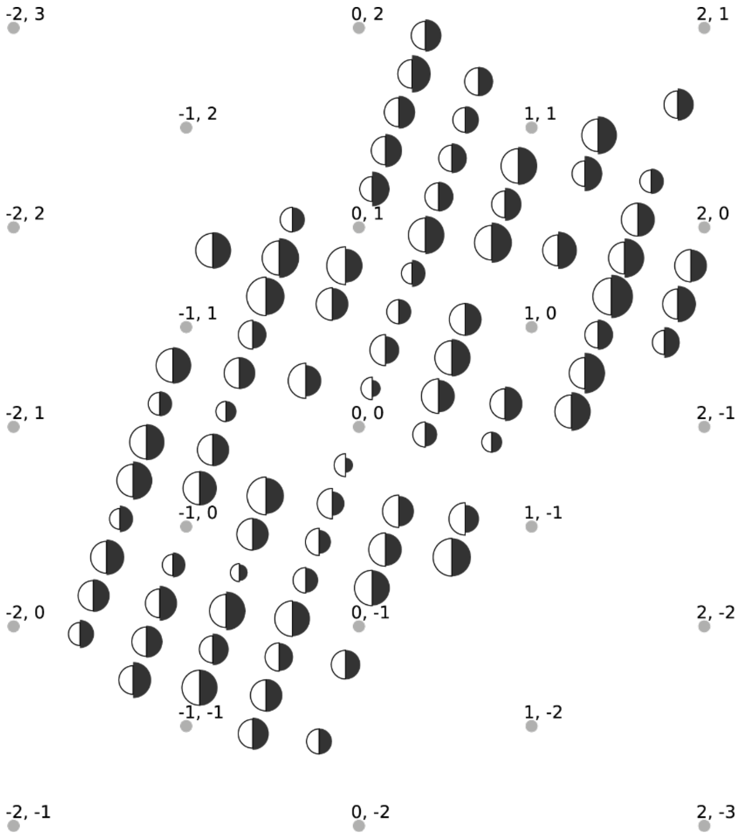

Figure S4 Comparison of the experimental (black semicircles) and predicted (open semicircles) in-plane structure factors of the full set of fractional order beams for the optimised SXRD structure shown in Figures 3(c) and (d) of the main paper. The areas of the semicircles are proportional to the measured and calculated structure factors

As described briefly in the main text, the influence of alternative lateral registries of the overlayer, relative to the outermost Au(111) layer, was explored, re-optimising the structural parameters for the best fit to the SXRD data for the Au adatom in atop, fcc hollow (atop third layer Au atoms), bridge and hcp hollow (atop second layer Au atoms). The chi squared values and the associated layer spacings for each registry are shown in Table S1, showing a clear preference for the Au adatom to occupy a local atop site relative to the outermost Au(111) layer.

Figure S6 shows a similar comparison of the measured in-plane structure factors with the results of ROD calculations for the best-fit no-adatom model, starting from the DFT no-adatom model but refining the structure in the same way as for the adatom model. The agreement is clearly poor, with predicted structure factors almost all too small. As reported in the main paper the chi-squared value for this model (for the complete experimental dataset of in-plane and rod scan measurements) was 6.611.

Table S1 Comparison of the chi-squared values and structural parameter values for fits to the SXRD data based on different lateral registry sites of the Au adatoms.

Adatom registry	Chi squared	Molecule height (Å)	Adatom height (Å)	Top layer rumpling amplitude (Å)	Second layer rumpling amplitude (Å)
atop	1.236	3.36	3.09	0.60	0.18
fcc hollow	1.350	3.12	3.02	0.58	0.21
bridge	1.691	3.45	1.76	0.55	0.22
hcp hollow	1.597	3.10	3.11	0.64	0.26

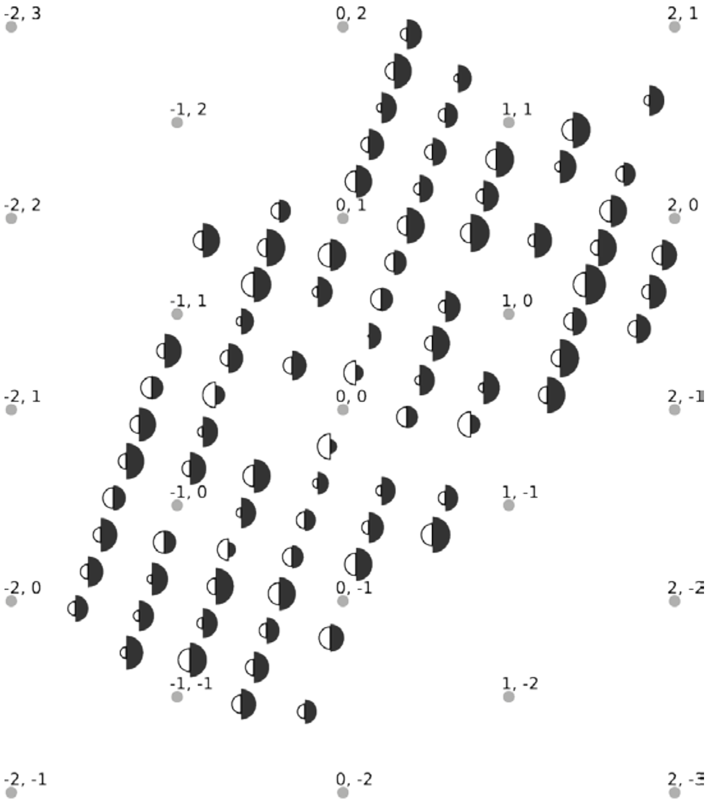

Figure S6 Comparison of the experimental (black semicircles) and predicted (open semicircles) in-plane structure factors of the full set of fractional order beams for the optimised no-adatom SXRD structure. The areas of the semicircles are proportional to the measured and calculated structure factors.

[1] Vlieg, E. ROD: a Program for Surface X-Ray Crystallography, *J. Appl. Cryst.* **2000**, *33*, 401-405.
